# Bioinformatics-based analysis reveals elevated CYTL1 as a potential therapeutic target for BRAF-mutated melanoma

**DOI:** 10.3389/fcell.2023.1171047

**Published:** 2023-09-06

**Authors:** Lei Tao, Yingyue Cui, Jiarui Sun, Yu Cao, Zhen Dai, Xiaoming Ge, Ling Zhang, Run Ma, Yunyao Liu

**Affiliations:** ^1^ Nanjing Institute for Food and Drug Control, Nanjing, China; ^2^ State Key Laboratory of Natural Medicines, School of Basic Medicine and Clinical Pharmacy, China Pharmaceutical University, Nanjing, China; ^3^ The Second Affiliated Hospital of Kunming Medical University, Kunming, China

**Keywords:** melanoma, BRAF mutations, CYTL1, molecular biomarker, cell migration and invasion

## Abstract

**Introduction:** Despite many recent emerging therapeutic modalities that have prolonged the survival of melanoma patients, the prognosis of melanoma remains discouraging, and further understanding of the mechanisms underlying melanoma progression is needed. Melanoma patients often have multiple genetic mutations, with BRAF mutations being the most common. In this study, public databases were exploited to explore a potential therapeutic target for BRAF-mutated melanoma.

**Methods:** In this study, we analyzed differentially expressed genes (DEGs) in normal tissues and melanomas, Braf wild-type and Braf mutant melanomas using information from TCGA databases and the GEO database. Subsequently, we analyzed the differential expression of CYTL1 in various tumor tissues and its effect on melanoma prognosis, and resolved the mutation status of CYTL1 and its related signalling pathways. By knocking down CYTL1 in melanoma cells, the effects of CYTL1 on melanoma cell proliferation, migration and invasion were further examined by CCK8 assay, Transwell assay and cell migration assay.

**Results:** 24 overlapping genes were identified by analyzing DEGs common to melanoma and normal tissue, BRAF-mutated and BRAF wild-type melanoma. Among them, CYTL1 was highly expressed in melanoma, especially in BRAF-mutated melanoma, and the high expression of CYTL1 was associated with epithelial-mesenchymal transition (EMT), cell cycle, and cellular response to UV. In melanoma patients, especially BRAF-mutated melanoma patients, clinical studies showed a positive correlation between increased CYTL1 expression and shorter overall survival (OS) and disease-free survival (DFS). *In vitro* experiments further confirmed that the knockdown of CYTL1 significantly inhibited the migration and invasive ability of melanoma cells.

**Conclusion:** CYTL1 is a valuable prognostic biomarker and a potentially effective therapeutic target in melanoma, especially BRAF-mutated melanoma.

## 1 Introduction

Cutaneous melanoma, which originates from melanin-producing skin melanocytes, is one of the most aggressive and difficult-to-treat human cancers. Over the past 50 years, its incidence has risen globally (Sung et al., 2021). The 5-year survival rate for patients diagnosed with metastatic melanoma is approximately 20%. It is characterized by the presence of mutations in multiple genes (Guo et al., 2021). BRAF mutations are the most predominant in melanoma development (Schadendorf et al., 2018). BRAF mutations are present in more than 50% of melanomas, with BRAFV600E being the most prevalent mutation type. This results in constitutive activation of RAF mitogen-activated protein kinase (MAPK) and extracellular signal-regulated kinase (ERK) signaling, which encourages melanoma proliferation and anti-apoptosis (Shen et al., 2020). Significant advancements in the treatment of melanoma have recently been made. However, because of its propensity for distant metastasis, poor prognosis, and frequent recurrence, melanoma remains a refractory illness. Thus, it is vital to research the molecular mechanisms behind melanoma formation and to look for efficient treatment targets.

A secreted protein containing 136 amino acids called cytokine-like 1 (CYTL1) was initially discovered in human CD34^+^ hematopoietic cells (Zhu et al., 2019). Since CYTL1 is highly expressed in cartilage tissues, such as the mouse inner ear and human articular cartilage, it helps maintain the homeostasis of these tissues and inhibits cartilage destruction in osteoarthritis (Shin et al., 2019; Zhu et al., 2020). CYTL1 has so far been demonstrated to have a variety of biological capabilities, including chemotaxis and pro-angiogenesis (Schneller et al., 2019; Xue et al., 2020). High levels of CYTL1 expression were found in tumor tissues and cell lines of human neuroblastoma, and silencing CYTL1 prevented the growth, migration, and invasion of neuroblastoma cells (Wen et al., 2012). In addition, CYTL1 is overexpressed and heavily methylated in human lung squamous cell carcinoma. Early in carcinogenesis, DNA methylation is linked to gene expression, and methylation of the promoter region silences genes. The hypermethylation of CYTL1 is consistent with its downregulation in SCC (Kwon et al., 2012). It has recently been shown that in breast cancer cells, CYTL1 is a tumor suppressor that keeps NDUFV1 stable and prevents metabolic reprogramming (Xue et al., 2022). According to the research mentioned above, different tumor types demonstrate varied expression patterns and roles for the cytokine CYTL1.

Bioinformatics analysis and gene expression profiling are helpful techniques to study the mechanisms of cancer development and reveal markers that predict patient prognosis (Chen et al., 2017; Peng et al., 2017). Reanalysis and integration of the vast quantity of data kept in public databases may yield fresh insights into the pathogenic processes underlying cancer. In this study, we analyzed the genes associated with BRAF mutations in the TCGA database. Comparing the distinct protein DEGs between melanoma and healthy tissues, overlapping DEGs were discovered. By building a protein-protein interaction (PPI) network and doing a functional enrichment analysis, we were able to pinpoint the gene CYTL1, which may be crucial to the emergence and development of melanoma with BRAF mutations.

By knocking down CYTL1 in BRAF-mutated melanoma cells, we found that CYTL1 has a negligible effect on melanoma cell proliferation but affects melanoma invasion and metastasis. In conclusion, CYTL1 may be a pro-oncogenic factor in melanoma. It could be a potential biomarker for diagnosis and prognosis, as well as a promising molecular target in BRAF-mutated melanoma.

## 2 Methods and materials

### 2.1 Microarray data

From the GEO database, we chose the profiles GSE46517 and GSE114445. 31 melanoma, nine nevus, and eight normal skin samples were included in GSE46517, which was built on the Agilent GPL96 platform (HG-U133A, Affymetrix Human Genome, U133A Array). The Agilent GPL570 platform-based GSE114445 (HG-U133 Plus 2, Affymetrix Human Genome U133 Plus 2.0 Array) contained 16 melanoma, seven dysplastic nevus samples, and six healthy skin samples. We studied the prognostic impact of CYTL1 in different tumors using the TIMER2.0 database (Li et al., 2020) and CYTL1 mutations and CNA in melanoma samples from 12 databases using the cBioPortal database (Cerami et al., 2012).

### 2.2 PPI network, GO, and KEGG pathway analysis of DEGs

We identified CYTL1 gene by protein-protein interaction (PPI) network construction and functional enrichment analysis. The STRING database (http://string-db.org) provides an objective analysis and vertical combination of protein-protein interactions (Szklarczyk et al., 2015). The PPI networks of DEGs were constructed with a combined score >0.4, and the network was visualized with Cytoscape (version 3.8.0).

The Gene Ontology (GO) and Kyoto Encyclopedia of Genes and Genomes (KEGG) pathway analyses of the DEGs were performed with DAVID (https://david.ncifcrf.gov/), which consists of integrated biological knowledgebase and analytic tools (Huang da et al., 2009). GO terms, and KEGG pathways with corrected *p*-value <0.05 were considered significantly enriched.

### 2.3 Expression level of DEGs and survival analysis

GEPIA (http://gepia.cancer-pku.cn/index.html) was used to analyze the RNA sequencing expression data for 471 cutaneous melanoma samples from the TCGA database and 1809 normal skin tissue samples from the GTEx database in accordance with a standard processing pipeline (Huang da et al., 2009). The fragments per kilobase per million reads (FPKM) values were calculated for gene quantification. Differential expression analysis of the control and PI groups was performed using DESeq2 (version 1.16.1), and genes with padj< 0.05 and |log_2_ (fold change)| > 1 were identified as DEGs. A volcano plot and a heatmap were generated with the ‘Ggpolt2’ and ‘ComplexHeatmap’ R packages.

### 2.4 Immune infiltration analysis

Tumor immune infiltration levels were determined using the single-sample gene set enrichment analysis (ssGSEA) method and the “GSVA” R package based on the TCGA-SKCM dataset (Bindea et al., 2013). Correlation analysis between CYTL1 and immune cell type infiltration was performed by Spearman rank correlation coefficient analysis. Charts and graphs were generated using the “ggplot2” R package (Tang et al., 2022). The correlation between CYTL1 expression and the relative number of tumor-infiltrating lymphocytes was calculated using the Tumor-Immune System Interaction Database website (TISIDB; http://cis.Hku.hk/TISIDB/) (Ru et al., 2019). *p*-values were determined by Spearman and Wilcoxon rank-sum tests.

### 2.5 Cell culture

The melanoma cell lines A2058 (RRID:CVCL_1059), A375 (RRID:CVCL_0132), M14 (RRID:CVCL_1395), and SK-MEL-28 (RRID:CVCL_0526) were purchased from the cell bank of the Chinese Academy of Sciences (Shanghai, China) and cultivated in DMEM media with 10% fetal bovine serum (FBS) and 1% penicillin/streptomycin. All experiments were performed with mycoplasma cells, and all cell lines were validated using STR (or SNP) profiling over the past 3 years.

### 2.6 Cell transfection

For small interference (siRNA) transfection, A2058 cells were seeded in 6-well plates in medium without antibiotics. After 24 h, 50 nmol/L of CYTL1 siRNA (siCYTL1) were used for gene silencing. Control cell transfection was performed with a Negative Control siRNA (siNC). Cell transfections were carried out with Lipofectamine RNAiMax (Invitrogen), following the manufacturer’s instructions. siCYTL1 and siNC were purchased from Sangon Biotech (Shanghai, China).

### 2.7 Western blot assay

RIPA buffer was used to obtain cell lysates, and Western blotting was performed as previously described (Liu et al., 2020), with primary antibodies raised against CYTL1 (Abcam) and GAPDH (Santa Cruz).

### 2.8 RT-qPCR assay

In the RT-qPCR assay, cells were lysed by RNA isolator (Vazyme, Nanjing, China, Cat. R401-01-AA). Following the manufacturer’s instructions, total RNAs were extracted, and the RT reagent kit (Vazyme, Nanjing) was used to reverse-transcribe them. The following primers were used in this study:

GAPDH-F:AATCCCATCACCATCTTCCA.

GAPDH-R:TGGACTCCACGACGTACTCA.

CYTL1-F: GAG​CCC​TCG​GAG​CCA​TGT.

CYTL1-R: AGG​ACC​GTA​GTC​ACT​GGG​AT.

### 2.9 CCK8 assay

The CCK-8 assay was performed to detect cell viability. 1000 cells per well in 96-well plates were used for inoculation. 10 µL of the CCK-8 solution was added to each well after 24 h, 48 h, and 72 h. The absorbance of each well was measured at 450 nm after the plates had been incubated for 1–4 h.

### 2.10 Wound healing assay

A2058 cells and A2058 cells that were knocked down of CYTL1 in the logarithmic phase were inoculated in a 6-well plate, and the experiment was carried out after the cells were completely attached. The surface of the monolayer cells was drawn in a straight line as the data before cell migration (0h). After 12 h and 24 h, observe the scratch repair and take photos. Randomly select the pictures of each group to calculate the cell migration efficiency.

### 2.11 Transwell assay

A2058 cells and A2058 cells that were knocked down of CYTL1 cells were diluted into 5×10^5^ cells/mL cell suspension in a serum-free medium. The transwell chambers were set up in a 24-well plate, and 400 μL of cell suspension and 600 μL of complete medium were added to the top and bottom chambers, respectively. Take the transwell chambers out of the culture after 24 h and use a cotton swab to remove any cells that have not made it through the microporous filter. The unmigrated cells were fixed with 100% methanol for 5 min, 0.5% crystal violet staining for 30 s, washed with water to remove the floating color and observed under the microscope. The fields (top, bottom, left, right, middle) randomly selected were taken pictures to calculate the average number of migratory cells.

### 2.12 Statistical analysis

Three duplicates of each experiment were carried out. GraphPad Prism was used to calculate the statistical analysis. Student’s t-test was used to assess the significance of the results between the two experimental groups, and one-way ANOVA was used to examine multiple group comparisons. Significant values included *p* < 0.05 (*), *p* < 0.01 (**), and *p* < 0.001 (***).

## 3 Results

### 3.1 Screening of potential targets in melanoma cells with BRAF mutation

**FIGURE 1:**
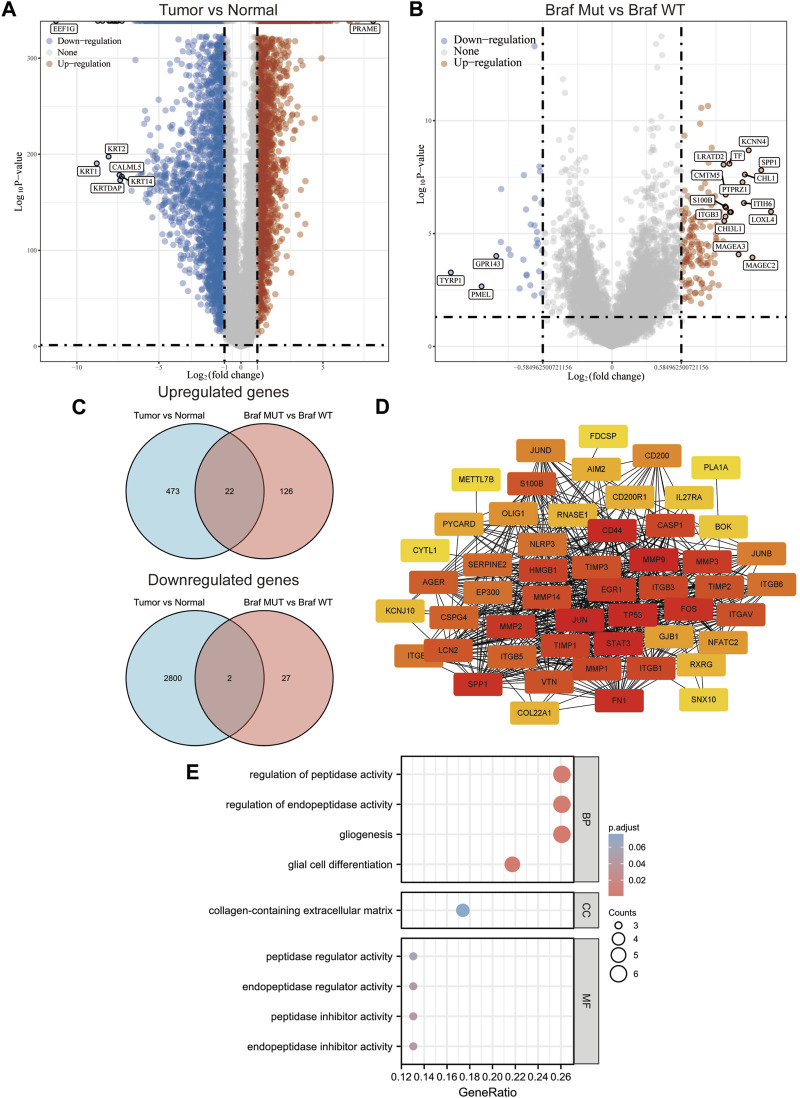
Screening of potential targets in melanoma cells with BRAF mutation. **(A,B)** Volcano plot of the differentially expressed genes in melanoma according to the TCGA dataset. **(C)** Venn of all overlapping DEGs. **(D)** Protein–protein interaction network of the overlapping DEGs. **(E)** Gene Ontology enrichment analyses of the overlapping DEGs.

A total of 471 cutaneous melanoma samples were included in the TCGA database, and 1809 normal skin tissue samples were included in the GTEx database. Analysis of the samples in both databases revealed that 495 up-regulated genes and 2802 down-regulated genes were identified (Figure 1A). Subsequently, we divided the cutaneous melanoma samples into BRAF wild and BRAF mutant types. Among them, 231 samples were BRAF wild type and 235 samples were BRAF mutant. We performed differential gene analysis and found 148 up-regulated genes and 29 down-regulated genes (Figure 1B). Among these differentially expressed genes (DEGs), 24 genes overlapped (Figure 1C). Among these overlapping DEGs, recognized tumor-associated genes such as TP53, JUN, MMPs, and STAT3 were located at the center of the STING protein interactions network (Figure 1D). We performed GO functional enrichment analysis to gain more insight into the screened DEGs. According to the GO analysis, the DEGs were primarily enriched in the following functions: regulation of peptidase activity, endopeptidase activity, gliogenesis, glial cell differentiation, and collagen-containing extracellular matrix (Figure 1E).

### 3.2 High expression of CYTL1 is associated with a poor prognosis of melanoma

**FIGURE 2:**
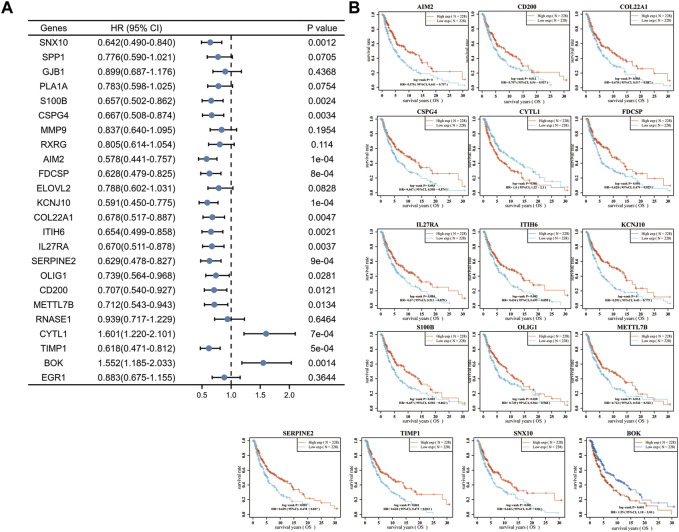
High expression of CYTL1 is associated with a poor prognosis of melanoma. **(A)** Forest plot of the *p*-value, risk coefficient (HR) and univariate analysis of the overlapping DEGs in melanoma. **(B)** Kaplan-Meier survival curves of SKCM patients with high expression or low expression of the overlapping DEGs.

To further validate the role of DEGs in melanoma, we examined the relationship between 24 DEGs and patient prognosis. SNX10, S100B, CSPG4, AIM2, FDCSP, KCNJ10, COL22A1, ITIH6, IL27RA, SERPINE2, OLIG1, CD200, METTL7B, and TIMP1 were found to be highly expressed in melanoma, yet negatively correlated with prognosis, i.e., the prognosis is better when the expression is higher. Increased expression of CYTL1 was associated with a poorer prognosis. Low expression of BOK in melanoma was positively correlated with prognosis, i.e., the prognosis is poorer when the expression is higher (Figure 2). These analyses showed that CYTL1 could be crucial to melanoma development.

### 3.3 High expression of CYTL1 is associated with poor prognosis in BRAF mutant melanoma

**FIGURE 3:**
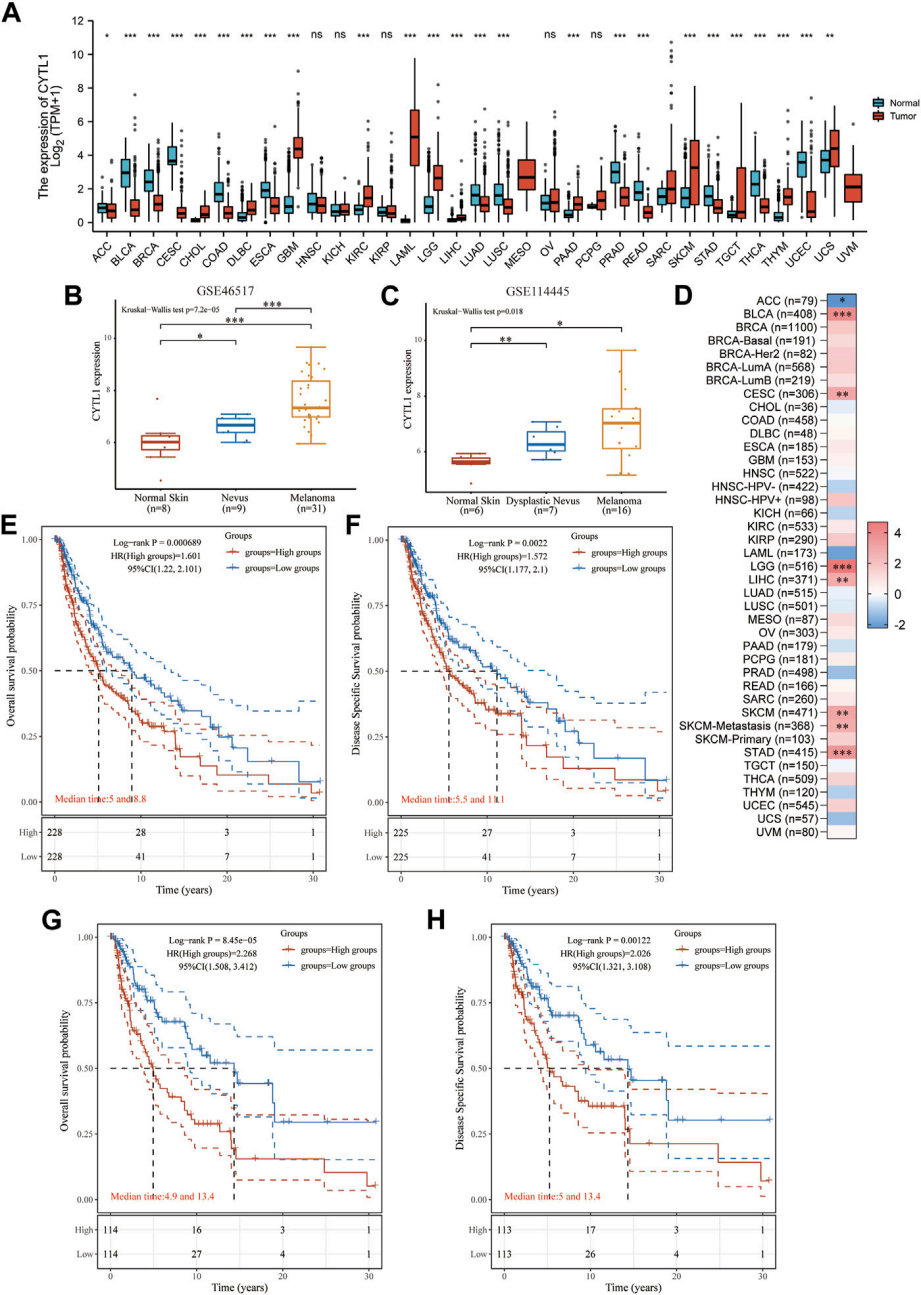
High expression of CYTL1 is associated with poor prognosis in BRAF mutant melanoma. **(A)**. CYTL1 expression levels in different tumor tissues and adjacent normal tissues from TCGA and GTEx databases. The expression level of CYTL1 in GSE46517 **(B)** and GSE114445 **(C,D)**. Heat map of the normalized coefficient of CYTL1 in Cox mode. Kaplan-Meier survival curves of OS **(E)** and DSS **(F)** in SKCM patients with high CYTL1 expression or low CYTL1 expression. Kaplan-Meier survival curves of OS **(G)** and DSS **(H)** in BRAF-mutant SKCM patients with high CYTL1 expression or low CYTL1 expression.

We further analyzed the differential expression of CYTL1 in different tumors and normal tissues. The results showed that CYTL1 was differentially expressed in a variety of tumors. CYTL1 was low expressed in ACC, BLCA, BRCA, CESC, COAD, ESCA, LUAD, LUSC, PRAD, READ, STAD, THCA, and UCEC, and highly expressed in CHOL, DLBC, GBM, KIRC, LAML, LGG, LIHC, PAAD, SKCM, TGCT, THYM, and UCS (Figure 3A). It is hypothesized that the primary factor influencing CYTL1 transcript levels is the kind of tumor. We next analyzed the expression of CYTL1 in two datasets (GSE46517 and GSE114445) in the GEO database. The results showed that CYTL1 showed a gradually increasing trend in normal skin, nevus, and melanoma (Figures 3B, C). Since the original data of these databases were from different sources, we used data from various sources to validate each other, which increased the authenticity and credibility of the conclusions.

Next, we analyzed the prognostic impact of CYTL1 in different tumors using the TIMER2.0 database. The results showed that CYTL1 significantly inhibited the survival of patients with BLCA, LGG, LIHC, SKCM, and STAD. In contrast, in ACC, CYTL1 acted as a tumor suppressor and prolonged the survival of patients (Figure 3D). Subsequently, we analyzed the OS and DFS of CYTL1 in cutaneous melanoma and BRAF-mutated melanoma. The results showed that CYTL1 was a pro-oncogenic factor in both melanoma and BRAF-mutated melanoma, and its high expression significantly suppressed patients’ OS and DFS (Figures 3E–H).

### 3.4 Genomic mutations in CYTL1 in melanoma

**FIGURE 4:**
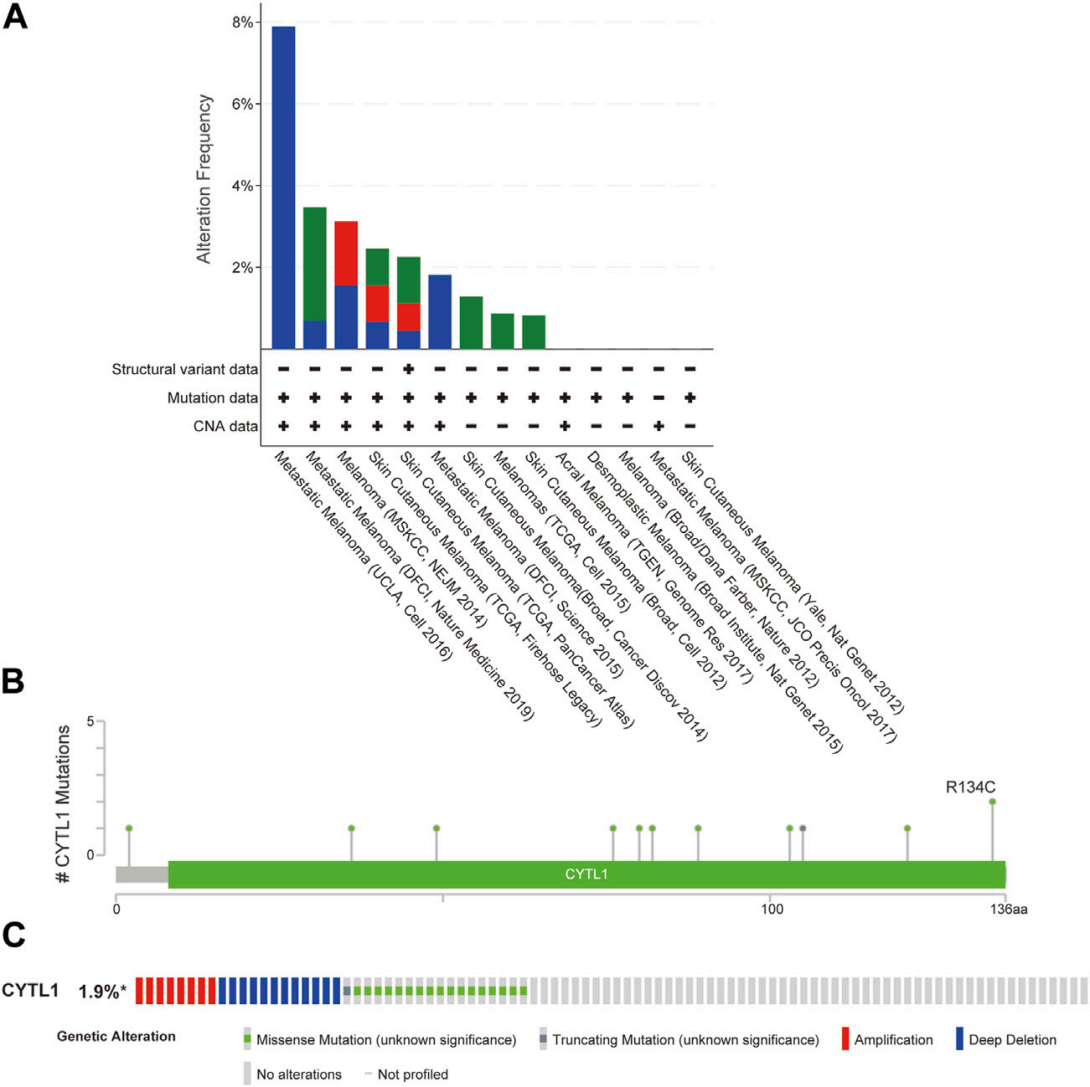
Genomic mutations in CYTL1 in melanoma. **(A)** Alteration frequency of CYTL1 in different data sets. **(B)** Mutation sites for CYTL1 gene mutations. **(C)** Type and frequency of CYTL1 gene mutations in SKCM.

We analyzed CYTL1 mutations and CNA in melanoma samples from 12 databases using the cBioPortal database. CYTL1 had different mutation frequencies in different datasets, 7.89% (UCLC, Cell 2016), 3.47% (DLCI, Nature medicine 2019), 3.13% (MSKCC, NEJM 2014), 2.46% (TCGA, Firehose Legacy), 2.25% (TCGA, PanCancer Atlas), 1.82% (DFCI, Science 2015), 1.28% (Broad, Cancer Discov 2014), and 0.87% (TCGA, Cell 2015), and 0.83% (Broad, Cell 2012) (Figure 4A). There were 11 mutated loci in the CYTL1 gene, with R134C being the most common (Figure 4B). About 1.9% of all patients had CYTL1 gene mutations (Figure 4C).

### 3.5 Analysis of genes and pathways associated with CYTL1 in melanoma

**FIGURE 5:**
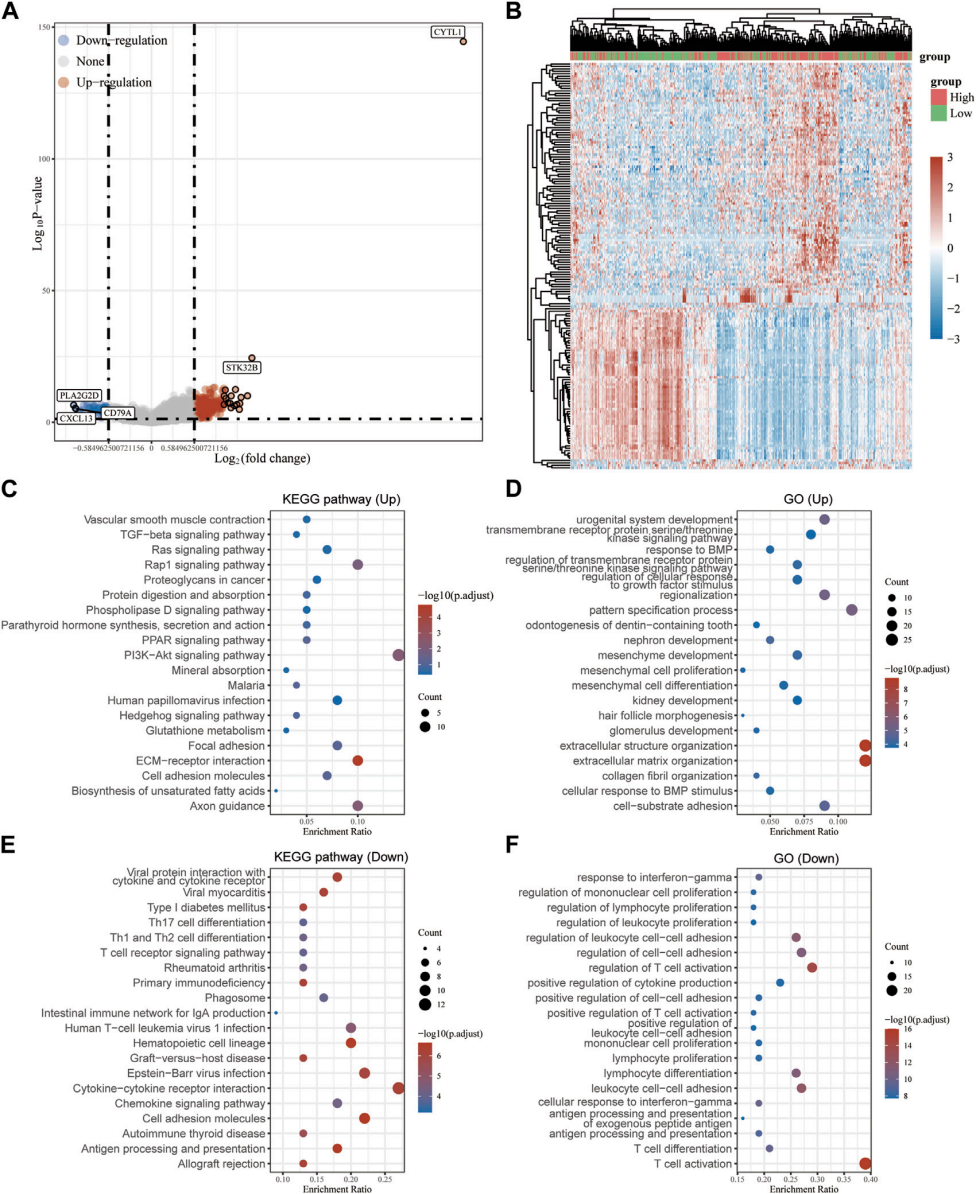
Analysis of genes and pathways associated with CYTL1 in melanoma. **(A)** Volcano plot of the differentially expressed genes in melanoma according to the TCGA dataset. **(B)** The heatmap of the differential gene expression. **(C–F)** GO and KEGG signaling pathways enrichment analyses of the DEGs.

To determine the biological function of CYTL1 in melanoma, we analyzed the differential genes in melanoma patients with high or low CYTL1 expression according to the median expression values of CYTL1 (Figures 5A, B). The KEGG pathway analysis revealed that CYTL1-associated up-regulated genes were mainly enriched in PI3K-Akt signaling pathway, Rap1 signaling pathway, ECM-receptor interaction, and Axon guidance (Figure 5C). CYTL1-associated down-regulated genes were enriched primarily on cytokine-cytokine receptor interaction, Epstein-Barr virus infection, cell adhesion molecules, immune cell differentiation, and chemokine signaling pathway (Figure 5E). GO functional enrichment analysis revealed that CYTL1-associated up-regulated genes were mainly enriched in extracellular structure organization, extracellular matrix organization, pattern specification process, cell-substrate adhesion, urogenital system development, and regionalization (Figure 5D). CYTL1-associated down-regulated genes were mainly enriched in T cell activation, leukocyte cell-cell adhesion, lymphocyte differentiation, and regulation of cell-cell adhesion (Figure 5F).

**FIGURE 6:**
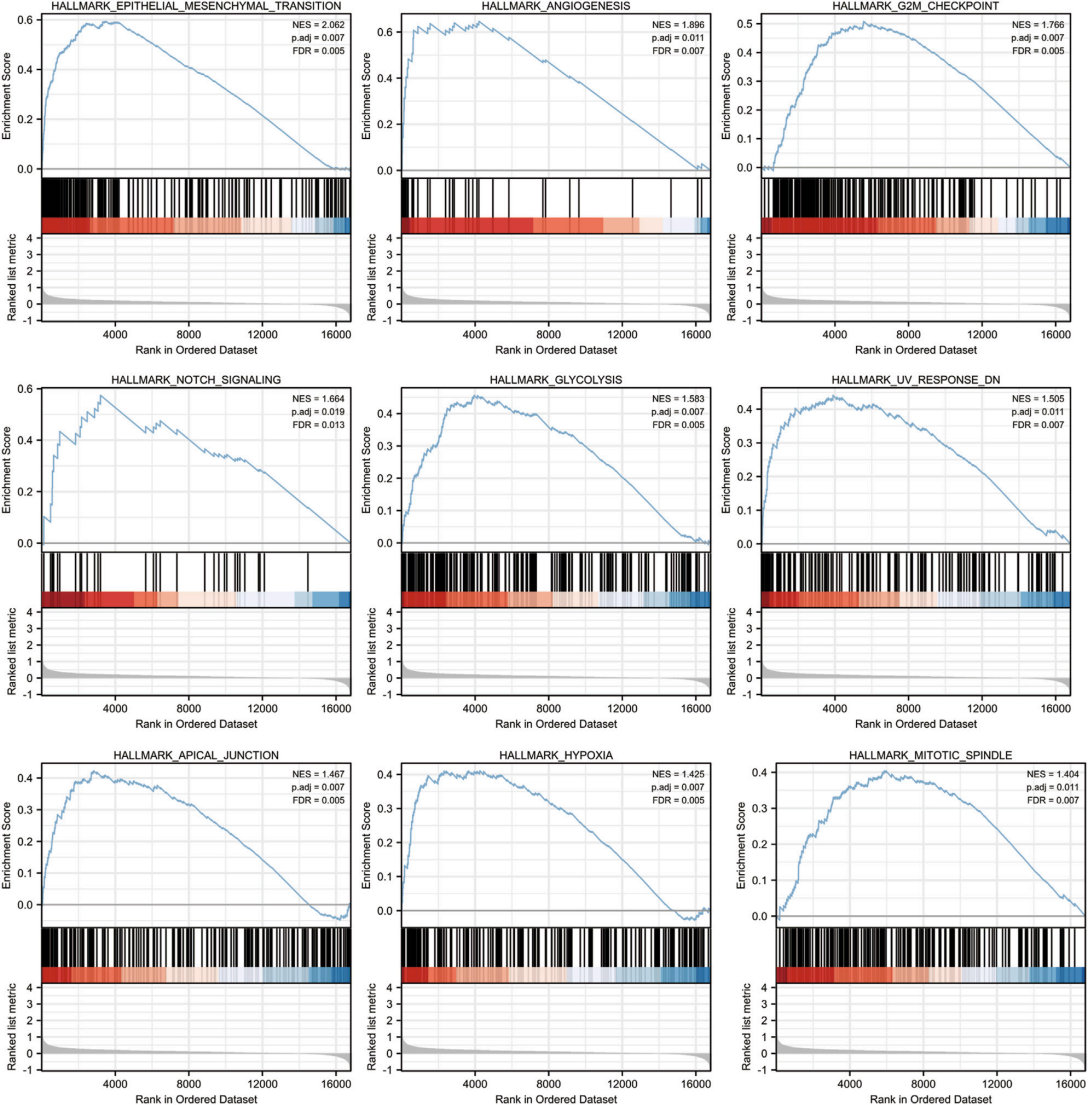
CYTL1-related signaling pathways in SKCM by GSEA software.

According to the results of a pathway study using gene set enrichment analysis (GSEA), CYTL1 plays a major role in the epithelial-mesenchymal transition (EMT), angiogenesis, G2M checkpoint, NOTCH signaling pathway, glycolysis, UV-related response, apical junction, and mitochondrial spindle assembly (Figure 6).

### 3.6 Correlation of CYTL1 expression with immune infiltration

**FIGURE 7:**
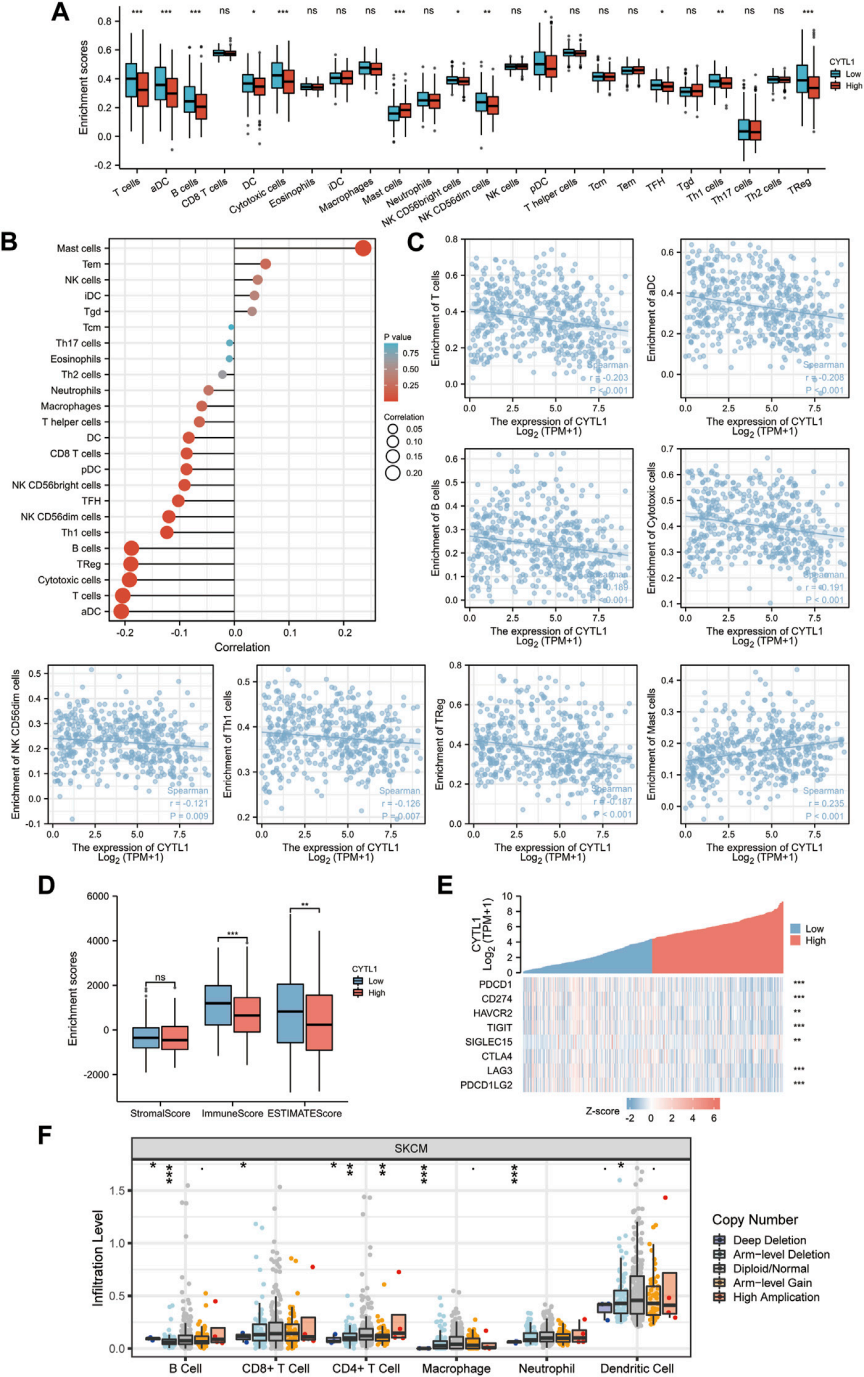
Correlation of CYTL1 expression with immune infiltration. **(A–D)** The correlation between CYTL1 expression and infiltration levels of immune cells. **(E)** Correlation analysis of CYTL1 expression and immune checkpoint-related genes in SKCM in the TCGA database. **(F)** Relationship between CYTL1 expression and SKCM tumor immune cell infiltration according to the TIMER database.

The prognosis of melanoma patients is impacted by tumor-infiltrating lymphocytes, which are critical to the development of cancer. The findings above imply that CYTL1 may be connected to immune-related pathways like T cell activation, cytokine production, cytokine receptor activation, and differentiation of Th17 cells (Figures 5C–F). Therefore, we next examined whether there is a relationship between CYTL1 and immune infiltration in melanoma. Our findings showed that mast cell numbers and CYTL1 mRNA levels showed a substantial and positive correlation. In contrast, CYTL1 expression was negatively correlated with T cells, aDC, B cells, DC, cytotoxic cells, NK CD56bright cells, NK CD56dim cells, pDC, TFH, Th1 cells, and Treg cells (Figures 7A–C). Meanwhile, we analyzed the relationship between CYTL1 and immune infiltration and stromal scores. The results showed that CYTL1 was not significantly correlated with the stromal component in the tumor microenvironment, while high CYTL1 expression significantly inhibited immune infiltration in the tumor microenvironment (Figure 7D).

Considering that CYTL1 may be a potential oncogene in melanoma, it was assessed how CYTL1 interacted with PDCD1, CD274, HAVCR2, TIGIT, SIGLEC15, CTLA4, LAG3, and PDCD1LG2. 8378574 (Figure 7E). These findings imply that CYTL1-mediated melanoma oncogenesis may entail tumor immune escape and anti-tumor immunity. In addition, the different copy statuses of CYTL1 affected immune immersion compared to normal tissues (Figure 7F).

### 3.7 Knockdown of CYTL1 inhibits migration and invasion of BRAF mutant melanoma cells

**FIGURE 8:**
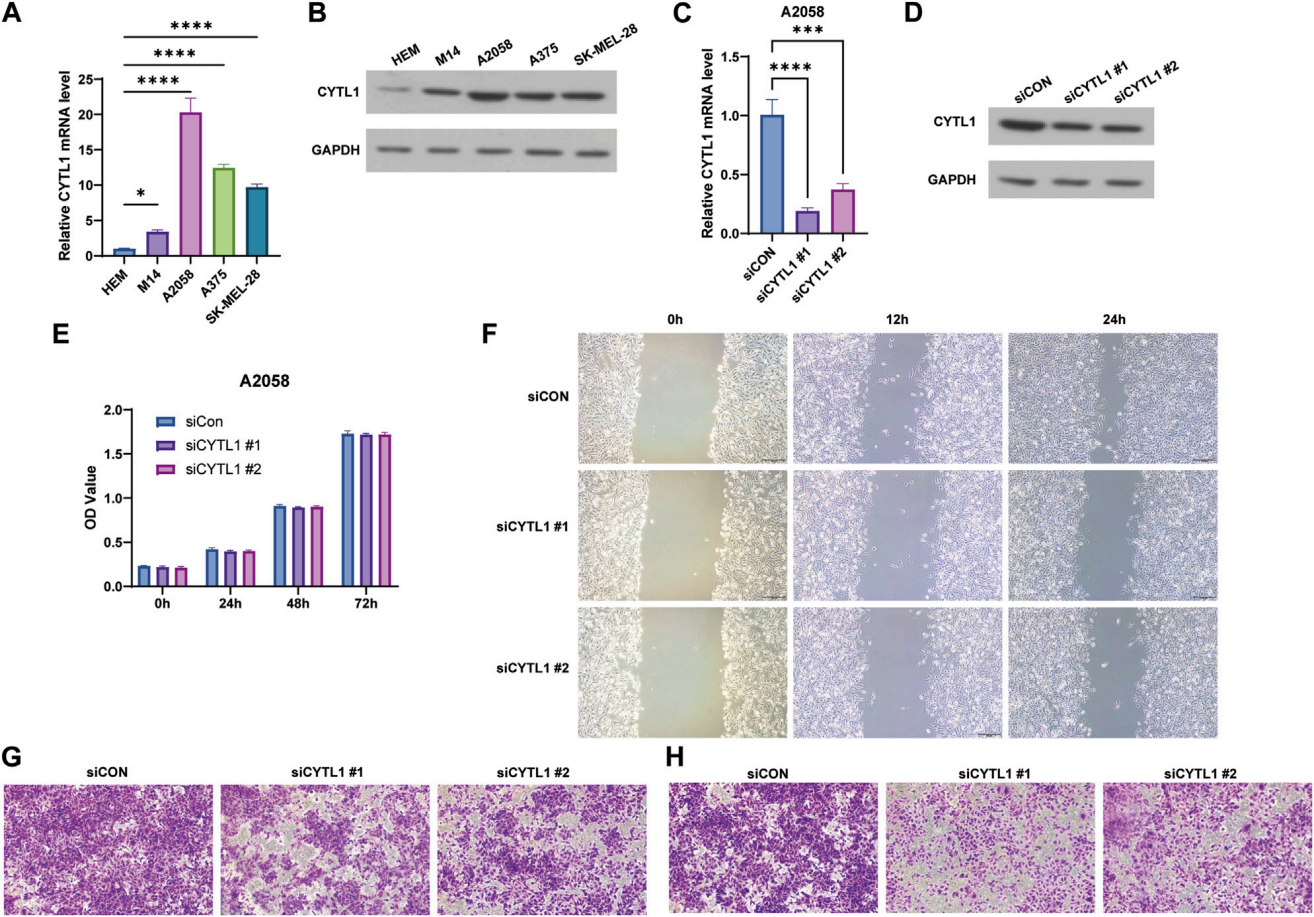
Knockdown of CYTL1 inhibits migration and invasion of BRAF mutant melanoma cells. **(A)** CYTL1 mRNA levels in different melanoma cells and HEM. **p ≤ 0.01, compared with HEM. **(B)** The protein levels of CYTL1 in different melanoma cells and HEM. **(C)** CYTL1 mRNA levels in A2058 cells transfected with si-CYTL1. **p ≤ 0.01, compared with siCon group. **(D)** The protein levels of CYTL1 in A2058 cells transfected with si-CYTL1. Cell viability **(E)**, wound healing **(F)**, cell migration **(G)** and cell invasion **(H)** of A2058 cells transfected with si-CYTL1.

Finally, we examined the expression of CYTL1 in human melanocytes HEM cells and melanoma cells A2058, A375, M14, and SK-MEL-28. Western blot assay and RT-qPCR experiments showed that the expression levels of CYTL1 in melanoma cells were all significantly higher than in HEM cells, among which A2058 cells had the highest CYTL1 levels (Figures 8A, B). Therefore, we selected A2058 cells for the follow-up experiment. Subsequently, we knocked down CYTL1 in A2058 cells to detect the effect of CYTL1 on the proliferation, migration, and invasion of melanoma cells (Figures 8C, D). CCK8 assay showed that CYTL1 had less impact on the proliferation of A2058 cells (Figure 8E). Wound healing assay and transwell migration assay showed that the knockdown of CYTL1 significantly inhibited the migratory ability of melanoma (Figures 8F, G). The transwell invasion assay with the addition of stromal gel showed that inhibition of CYTL1 expression significantly inhibited the invasive ability of A2058 cells (Figure 8H). This result is consistent with the positive correlation between CYTL1 and EMT in Figure 6. Taken together, CYTL1 can affect the invasive metastasis of A2058 cells.

## 4 Discussion

A significant development in the current management of metastatic melanoma is the identification of BRAF V600E as a therapeutic target. Between 35 and 50 percent of melanomas have active BRAF mutations, which encourage tumor growth *via* the MAPK/ERK signaling pathway (Czarnecka et al., 2020). BRAF inhibitors alone or in combination with MEK inhibitors, for example, significantly boosted OS and PFS in melanoma patients (Perreault et al., 2019). However, with the development of acquired drug resistance, patients’ sensitivity to the drugs began to decline, and chronic treatment with these inhibitors encountered challenges. Therefore, analyzing the presence of genes that can act synergistically after BRAF mutations and developing their targeting agents are essential research directions to address drug resistance. The results of this study support that CYTL1 may be considered a newly discovered biomarker for the diagnosis, prognosis, and treatment of melanoma. Secretory CYTL1 enhances MAPK/ERK pathway activation through C-C chemokine receptor type 2 in chronic granulocytic leukemia (CMML) (CCR2). By causing leukemic monocytes to undergo apoptosis, inhibition of CYTL1 in conjunction with MEK inhibitors can halt the course of CMML (Sevin et al., 2021). The results of this study and our study suggest that the same treatment modality may be tried for melanoma.

In this study, we analyzed the TCGA database of melanoma versus normal tissue, BRAF mutated melanoma versus BRAF wild-type melanoma and obtained a total of 24 differential genes, i.e., 22 up-regulated differential genes and two down-regulated differential genes, including TP53, MMPs, and other recognised melanoma oncogenes (Weiss et al., 2022). Interestingly, only CYTL1 expression was shown to be adversely connected with patient OS when we looked at the association between these 24 genes and the OS of melanoma patients. By analyzing the GSE46517 and GSE114445 datasets, we found that CYTL1 expression was progressively upregulated in normal skin, nevi or malignant nevi, and melanoma. We also validated CYTL1 expression in melanoma cells and normal melanocytes by RT-qPCR assay and found that in melanomatous cells, compared to normal cells, CYTL1 mRNA expression was considerably higher, indicating that CYTL1 may be a potential diagnostic target with excellent sensitivity and specificity.

The human CD34^+^ cells seen in bone marrow and umbilical cord blood were where CYTL1 was first discovered. CYTL1 possesses a signal peptide at its N-terminal end, from amino acid (aa) residues one to aa22, which is indicative of a secreted protein with a fold like that of the chemokine interleukin (IL)-8, according to bioinformatic analysis (Ai et al., 2016). High levels of CYTL1 expression have been seen in tumor tissues and cell lines from human neuroblastoma, and inhibiting CYTL1 prevents SH-SY5Y neuroblastoma cells from proliferating, migrating, and invading (Wen et al., 2012). In gastric cancer, CYTL1 has also been shown to be a necroptosis-promoting oncogene (Khan et al., 2022). By reducing STAT3 phosphorylation, CYTL1 prevents lung cancer tumor spread (Wang et al., 2019). A recent study reported that intracellular CYTL1 is a potential tumor suppressor that stabilizes NDUFV1 to prevent metabolic reprogramming in breast cancer (Xue et al., 2022). However, in our study, CYTL1 was a pro-oncogenic factor in melanoma, and in melanoma patients, its elevated expression was linked to a shorter OS and DFS., especially those with BRAF mutations. Knockdown of CYTL1 inhibited the migration and metastasis of melanoma. The different roles of CYTL1 in various tumors deserve to be explored in depth.

In conclusion, CYTL1 expression is significantly upregulated in melanoma, and its upregulation can promote EMT in melanoma cells and promote melanoma progression. High levels of CYTL1 are a poor prognostic factor for melanoma patients and can also be a potential therapeutic target for BRAF-mutated melanoma.

## Data Availability

The original contributions presented in the study are included in the article/supplementary material, further inquiries can be directed to the corresponding authors.
